# Supplementary material for: NeuDiff Agent: a governed AI workflow for single-crystal neutron crystallography
Source: J Appl Crystallogr. 2026 Jun 24;59(Pt 4):1102–11. doi: 10.1107/S1600576726004474 (PMC13440046; doi:10.1107/S1600576726004474)
Supplement: Supplementary file 1 [file j-59-01102-sup1.pdf]

JOURNAL OF
APPLIED
CRYSTALLOGRAPHY

Volume 59 (2026)

Supporting information for article:

NeuDiff Agent: a governed AI workflow for single-crystal neutron crystallography

Zhongcan Xiao, Leyi Zhang, Guannan Zhang and Xiaoping Wang

Supporting Information

NeuDiff Agent: A Governed AI Workflow for Single-Crystal Neutron Crystallography

Zhongcan Xiao [a], Leyi Zhang [a,b], Guannan Zhang [c], Xiaoping Wang [a,*]

[a] Neutron Scattering Division, Oak Ridge National Laboratory, Oak Ridge, Tennessee 37831, USA

[b] Department of Linguistics, University of Illinois Urbana-Champaign, Urbana, IL 61801, USA

[c] Computer Science and Mathematics Division, Oak Ridge National Laboratory, Oak Ridge, Tennessee 37831, USA

S1. TOPAZ data reduction and analysis workflow

S1.1. Reduction definition and execution

TOPAZ single-crystal neutron TOF datasets were reduced from event-mode NeXus files on the SNS analysis environment using the TOPAZ ReductionGUI as the control interface for the standard Mantid-based TOPAZ reduction scripts. NeuDiff Agent assists users prior to execution by validating and suggesting ReductionGUI inputs; the GUI then writes a single text configuration file (*.config*) that fully defines the reduction instance and serves as the provenance record for deterministic reruns.

When reduction is launched, the GUI executes the TOPAZ reduction scripts under the recorded parameter set, performing peak finding, indexing and UB determination (or UB reuse when provided), peak integration in reciprocal space, wavelength-dependent normalization using the vanadium-derived incident spectrum, Lorentz corrections, and absorption correction using ANVRED as configured.

S1.2. Required configuration inputs (parameter classes)

Data selection and instrument inputs: IPTS identifier, run list (and any grouping by crystal setting), calibration/background inputs, detector masks, and optional UB matrix (*.mat*) for initialization or reset.

Resolution and spectrum bounds: wavelength window and resolution limits (e.g. d_min or equivalent bounds), and the vanadium-derived incident spectrum file used for wavelength-dependent normalization.

Peak finding, indexing and UB handling: peak-selection thresholds, indexing tolerances, and criteria for accepting a stable UB with physically plausible lattice parameters.

Integration model settings: peak and background region definitions (peak radius and background inner/outer boundaries) and any configured rejection criteria.

Absorption treatment: ANVRED model selection (spherical or polyhedral) and required sample descriptors (chemical formula, Z, unit-cell volume, and size parameter or polyhedral face definitions).

S1.3. Outputs and acceptance checks

Reduced data were exported as wavelength-resolved SHELX HKLF 2 reflection files with batch identifier and wavelength recorded per reflection. Symmetry averaging was not applied at the reduction stage to avoid conflating wavelength-dependent systematic effects in TOF Laue data. Where configured, an additional HKLF 2 file was generated using automated outlier rejection (e.g. a Z-score threshold) to enable sensitivity tests without manual editing of reflection lists.

A reduction was considered acceptable when indexing yielded a stable UB matrix with physically plausible lattice parameters, indexed-peak statistics met the preset thresholds, and integration produced defensible reflection statistics within the stated resolution limits without dominant failure signatures in the Mantid reduction logs. If these criteria were not met, reprocessing was performed only via controlled updates captured in a newly saved configuration file.

S1.4. Refinement and validation linkage

HKLF 2 outputs were used for standard neutron structure refinement and subsequent CIF generation and checkCIF validation as described in the main manuscript. CIF validation is treated as an explicit proofing stage; corrective edits are recorded in an auditable change log.

Current scope and limitations

The benchmark reported in this manuscript is limited to structural crystallography on TOPAZ for a periodic nuclear structure. The governed workflow presently covers reduction setup,

execution of the standard reduction pipeline, refinement support, hydrogen completion, and CIF proofing. Magnetic structure determination, magnetic symmetry analysis, and incommensurate or superspace refinement were not benchmarked here and will require additional tool wrappers and validation gates in future versions.

S2. LLM backends and decoding parameters

S2.1. Models evaluated

NeuDiff Agent is designed to be model-agnostic as a prototype: the orchestration logic, memory, RAG document set, and tool wrappers are identical across LLMs. In this work, we evaluated two representative LLM backends:

Online State-of-the-Art (SOTA) model, accessed via web APIs:

Gemini 3.0 Pro

Locally-hosted on-premises open-weight model, served via Ollama on ORNL HPC systems:

GPT-OSS 120B

Across models, prompts, state representation, retrieval document set, tools, and verification gates were held constant. Only the LLM backend changed.

S2.2. Decoding parameters and reproducibility controls

Decoding parameters were fixed within each backend across all repeated runs (Table S1). Temperature was set to T = 1.0 for both backends to avoid per-run tuning and to maintain consistent interaction behavior. Reproducibility at the workflow level is enforced primarily by deterministic tool execution, explicit parameter validation, and state-gated progression through reduction, refinement, and validation stages, rather than by relying on any single sampling setting. Robustness is therefore evaluated empirically across repeated end-to-end runs, as described in the benchmark protocol below.

Table S1 LLM backends, hosting mode, and decoding parameters used in the benchmark.

Model	Hosting mode	Temperature	Top-K	Top-P	Context window size
GPT-OSS 120B	DGX server	1.0	40	0.9	131072 (combined)
Gemini 3.0 Pro	Online	1.0	64	0.95	1 M (in) / 64 K (out)

S3. RAG framework with semantic chunking

S3.1. Corpus construction

NeuDiff Agent uses a retrieval-augmented generation (RAG) framework to answer questions that require up-to-date or facility-specific information beyond the LLM's pre-training window. The RAG corpus includes:

- TOPAZ instrument web pages and documentation hosted at ORNL/SNS;
- beamline-specific FAQs, run-preparation notes and user guides;
- selected neutron-diffraction references covering data reduction, refinement and sample environments;
- facility-approved HOWTO documents for practical issues such as file paths, sample-environment modes, and connection instructions to SNS analysis nodes.

Source documents are ingested offline into a local corpus using a facility-controlled ingestion job that mirrors approved web pages and documentation while preserving headings and section structure. Ingestion is performed on a controlled schedule and produces a versioned knowledge-base release that is immutable once published; optional signing can be used to support integrity checks. Runtime retrieval is constrained to a user-selected local release and does not perform live crawling. For every retrieved chunk, the agent records provenance fields including release version, source identifier, URL, retrieval timestamp, and section heading in the audit directory to support traceability and exact replay. The retrieval corpus is strictly separate from user chat logs and experimental data; user data are never added to the knowledge base.

S3.2. Embedding model and document representation

To support semantic search, each document is divided into chunks and encoded into fixed-length vectors using a MiniLM-based sentence-embedding model (Wang *et al.*, 2020). In the current implementation:

- A general-purpose MiniLM model (version 2) is used as the encoder.
- Each chunk is mapped to a 384-dimensional embedding vector capturing its semantic content.
- Embeddings are stored in a ChromaDB vector database (version 1.2.1).

The embedding model is treated as a modular component; different sentence-transformer variants can be substituted without changing the rest of the RAG pipeline, provided they output fixed-size vectors.

S3.3. Chunking strategy and retrieval hyperparameters

In practice, the chunking strategy strongly affects retrieval quality. We therefore adopt a semantic chunking scheme tuned for instrument documentation:

Raw HTML / Markdown documents are first cleaned and segmented by headings and logical sections.

Text is then split into overlapping windows based on character count, preserving paragraph boundaries where possible.

In the configuration used for the results reported in the main text:

Chunk window size: 1600 characters

Overlap between adjacent chunks: 200 characters

Indexing: all chunks are embedded and stored in ChromaDB with their source document ID, heading, and character offsets.

At query time:

The user's question is embedded with the same MiniLM encoder.

A similarity search retrieves candidate chunks from the vector index based on cosine similarity.

Top-K semantic retrieval: the top 60 chunks by cosine similarity are retrieved as candidates.

These candidates are re-ranked with BM25 (Robertson & Zaragoza, 2009) to boost exact keyword matches and penalize overly generic chunks.

The highest-ranked chunks within a token budget are concatenated, along with their headings and short provenance notes, and injected into the LLM prompt for answer generation.

The chosen window size and overlap reflect a trade-off: smaller chunks maximize precision but risk losing context; larger chunks preserve context but can introduce irrelevant

information and increase token usage. Empirically, the 1600/200/60 configuration provided a good balance for TOPAZ documentation.

Figure S1 provides a representative example illustrating why semantic chunking was selected for facility documentation: it aggregates related information across sections so that retrieval returns a complete context window for generation.

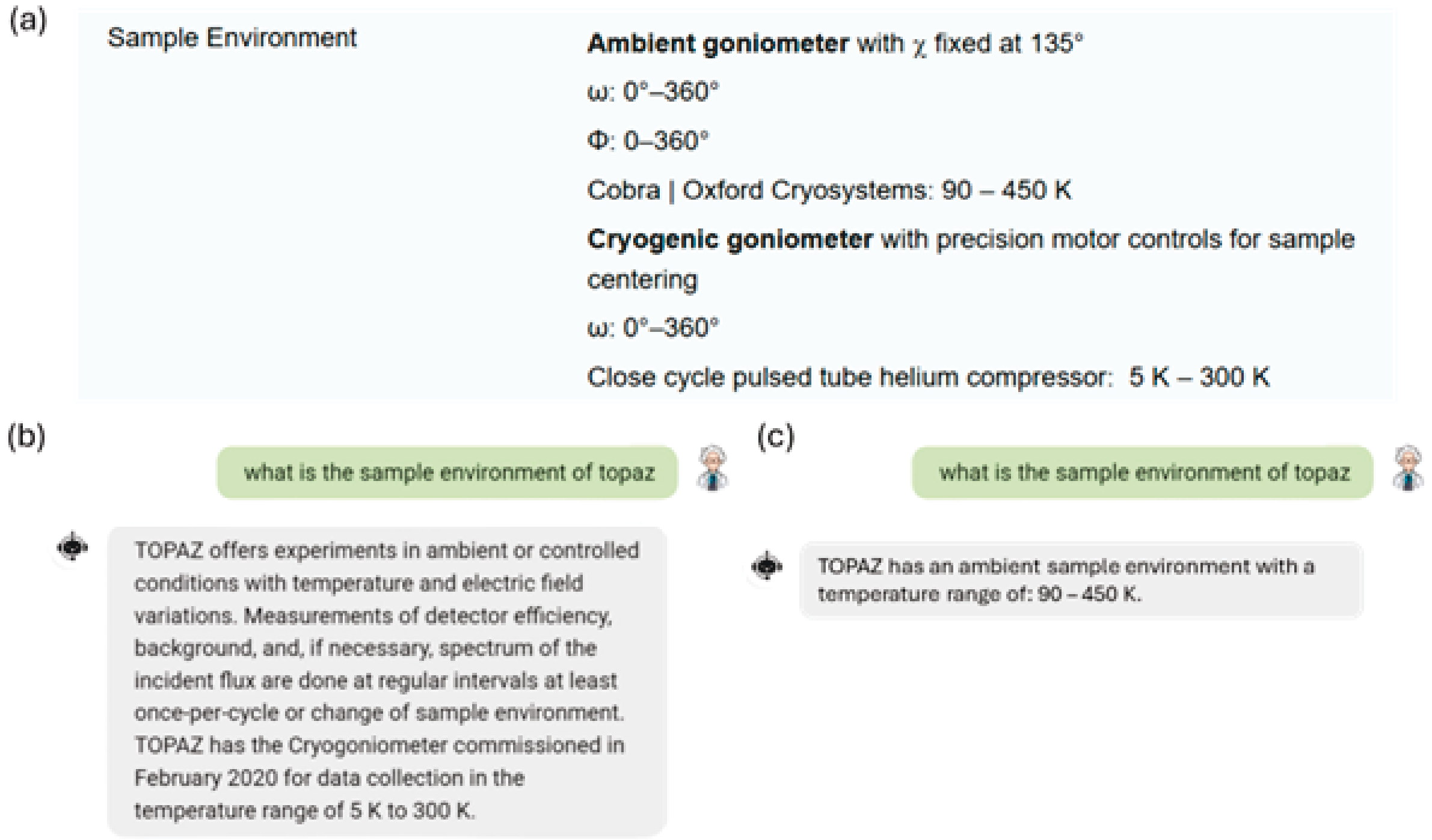

Figure S1 Comparison of retrieval outputs under different chunking strategies. (a) Source excerpt from an ORNL TOPAZ documentation page as stored in the local knowledge-base release. (b) Agent response using RAG with semantic chunking based on MiniLM embeddings. (c) Agent response using RAG with sentence-splitting chunking. Semantic chunking captures both ambient and non-ambient sample environments in a single response by aggregating related information across sections, whereas sentence-based splitting tends to return only the ambient environment and omits relevant details dispersed across multiple sections.

S3.4. Comparison with sentence-based chunking

To evaluate the benefit of semantic windowing, we compared:

1. Semantic chunking with MiniLM embeddings (as above), and
2. A simpler sentence-splitting scheme, where documents were split at sentence boundaries without overlapping windows.

For a representative TOPAZ sample-environment query, the sentence-splitting scheme often returned only the ambient environment because relevant non-ambient modes were described in separate sections. In contrast, semantic chunking tended to retrieve both ambient and non-ambient environment descriptions within the retrieved context window (Fig. S1b,c).

S4. Agent constraints, verification gates, and provenance bundle

S4.1. Terminology and operational definitions

The following terms are used consistently in the main text and Supporting Information.

Verification gate: a deterministic check executed before accepting a workflow state transition (for example, schema validation for configuration inputs, parsing reduction and refinement logs for known failure signatures, or checkCIF alert thresholds).

Fail-closed: if a verification gate fails, the workflow does not advance. The agent surfaces the failure, proposes a bounded corrective action, and requires explicit user authorization before re-execution.

Intervention event: a user-authorized corrective action taken to clear a failed gate (for example, updating ReductionGUI parameters, modifying refinement inputs, or correcting CIF metadata).

Audit directory: the per-run directory that records the complete tool-call trace (arguments, outputs, status codes), timestamps, gate outcomes, and generated artifacts required for controlled replay and facility oversight.

Governance: enforceable constraints on agent actions coupled with complete provenance. In NeuDiff Agent, governance is implemented through tool allowlisting, explicit authorization checkpoints, fail-closed gates at workflow transitions, and replayable audit logs.

S4.2. Determinism and logging

NeuDiff Agent is designed for auditability and replay as a research prototype. To support reproducibility:

- Stochastic preprocessing (for example, embedding settings) is fixed per deployment.
- For each run, the following are snapshotted into a per-run directory:
  - system prompt and key configuration
  - neutron knowledge schema summary
  - complete user-agent dialogue
  - all tool-call arguments and tool outputs (including exit codes and warnings)
  - timestamps and run identifiers enabling reconstruction of the full analysis path

Secrets (SSH keys, API tokens) are excluded from logs.

S5. Security and data governance

SSH and LLM API credentials are stored in environment variables on the workstation and are not injected into the agent prompt.

Remote execution is restricted to SNS analysis file systems and user-owned directories.

Access to models hosted on the DGX server is secured via SSH tunneling with user credentials.

The retrieval knowledge base is stored locally as versioned, immutable releases with original URLs and timestamps recorded; refresh operations are performed offline on a controlled schedule under facility governance.

Raw scientific data remain on SNS storage; only derived artifacts explicitly requested by the user (for example, configuration files, HKL files, CIFs) are exposed through the UI for download.

S6. Runtime environment, determinism and governance

This Supporting Information documents the runtime environment, determinism controls, and governance and logging mechanisms that underpin NeuDiff Agent as a facility research prototype, and it provides the task definitions and timing protocol used to quantify end-to-end acceleration in the prototype evaluation.

S6.1. HPC environment for model hosting

Selected LLMs were served from an ORNL SNS DGX server using Ollama. Node configuration:

Operating system: Ubuntu 22.04

Memory: 1 TB RAM

CPUs: AMD EPYC 7742 processors (4)

GPUs: NVIDIA A100 (8)

LLM serving: Ollama (models hosted on GPUs; NeuDiff Agent communicates via internal network APIs)

This configuration supports on-premises hosting of open-weight models while maintaining low-latency interactive access for the prototype evaluation.

S6.2. Analysis workstation environment

The NeuDiff Agent orchestration layer, UI, and agent logic ran on a workstation with:

Operating system: Ubuntu 22.04

Python: 3.11

Mantid: 6.10

SHELXL: 2019/3

ChromaDB: 1.2.1

LangGraph: 0.6.4

Trame: 3.10

CPU: Intel Core i5-13600H

RAM: 128 GB

All case-study runs reported in the main text used CPU-only execution for agent components on this workstation. For data access and execution of Mantid scripts, SHELXL, VESTA, and PLATON, the agent connects to ORNL SNS analysis nodes via SSH under existing facility policies.

S7. Controlled prompt list and replay assets

S7.1. Fixed prompt list used in the benchmark

The benchmark used a fixed prompt sequence to standardize user intent and reduce evaluator degrees of freedom. The prompt list is reproduced below exactly as used.

1. Hi
2. Introduce yourself
3. Introduce the workflow of neutron data processing that you can provide guidance with

4. I want to perform the data processing task
5. What is Z factor, and how to calculate it
6. How do I find the crystal system and other symmetry properties
7. Where is the calibration file
8. What is run number
9. What run numbers should I include in this setup
10. What format is accepted for run numbers
11. What is background file
12. What is ub file
13. What is ub matrix
14. How to calculate minimum d-spacing
15. How to determine maxQ
16. What is split threshold
17. What values of split threshold is recommended to use
18. Where would the data be stored
19. What is indexing tolerance
20. Explain Width of border in which peaks are rejected
21. What is Predict Peaks
22. What is Specific Peak Size
23. What is satellite peaks
24. How do I mask a satellite peak for my sample
25. Where is spectrum file located
26. What is reciprocal lattice and scale factor
27. What is Minimum integrated intensity
28. What is logfile
29. What is statistics of peaks

30. How should I choose the statistics metrics
31. What is minimum/maximum wavelength
32. Run reduction
33. Analyze reduction results
34. What is refinement
35. Continue to refinement step
36. Analyze refinement results
37. What is the error
38. How to solve the error
39. Improve the input file accordingly
40. Rerun the calculation
41. Summarize the results
42. Check cif file
43. How to resolve the alerts
44. Rerun calculation
45. Proof cif file

S7.2. Task definition and timing protocol

To quantify end-to-end acceleration, benchmark runs were segmented into two states aligned to the scientific workflow:

Stage1, Setup and run calculation: configuration preparation and validation; execute reduction and refinement computations.

Stage 2, Iterate and proof results: interpret results and iterate as needed; publication validation (including checkCIF loop).

Timing was recorded as wall time, namely elapsed time from task start to task completion.

Machine time: LLM latency (response generation plus tool-call planning) plus deterministic tool runtime (Mantid, SHELXL, parsing, plotting, checkCIF submission and report retrieval).

Human time: wall time minus machine time (reading, decision-making, manual edits).

Reference manual timing (435.0 min) was measured by executing the full analysis workflow without NeuDiff Agent, using the same workstation and software environment used for the agent-assisted runs. This reference provides the baseline for quantifying end-to-end acceleration. Timing started at the beginning of the workflow step and ended when a validated CIF and publication-facing crystallographic package were produced.

S7.3. Refinement statistics

Details of refinement fitting parameters, crystallographic statistics, and H-bond geometry are given in Tables S2-S4

Table S2 Refinement summary statistics and reflection counts for the scolecite case study.

Condition	R1	wR2	GoF (S)	Nref (independent reflections)	N used in R1	Theta(max) (deg)	Notes
Before agent optimization (1st CIF)	0.1846	0.4594	2.195	6416	6382	77.909	Direct comparison baseline reported in the main manuscript;
After agent optimization (validated final CIF)	0.0554	0.1297	1.062	8474	8441	78.439	checkCIF summary reports no level A or B alerts after validation

Table S3 Key crystallographic statistics for the final proofed and validated CIF (scolecite case study).

Crystal data	
Sample	scolecite
Chemical formula	$Al_4O_{20}Si_6 \cdot 6(H_2O) \cdot 2(Ca)$

M_r	784.72
Crystal system, space group	Monoclinic, *Cc*
Temperature (K)	100
a, *b*, *c* (Å)	6.5129 (1), 18.9377 (2), 9.7582 (1)
β (°)	108.958 (1)
V (Å^3)	1138.28 (3)
Z	2
Radiation type	Neutrons, λ = 0.600 Å
μ (mm^{-1})	0.0668 + 0.040618 λ (λ in Å)
Crystal size (mm)	1.63 × 1.19 × 0.62
Data collection	
Diffractometer	TOPAZ
Absorption correction	For a sphere<br>C. W. Dwiggins, Jr., Acta Cryst. A31, 395 (1975).
T_{min}, T_{max}	0.817, 0.931
No. of measured, independent and observed [$I > 2\sigma(I)$] reflections	23974, 8474, 8441
R_{int}	0.099
(sin θ/λ)$_{max}$ (Å^{-1})	1.633
Refinement	
$R[F^2 > 2\sigma(F^2)]$, $wR(F^2)$, S	0.055, 0.130, 1.06
No. of reflections	8474
No. of parameters	227
No. of restraints	2
H-atom treatment	All H-atom parameters refined
$\Delta\rho_{max}$, $\Delta\rho_{min}$ (fm Å^{-3})	1.41, −1.76
Absolute structure	All f'' are zero, so absolute structure could not be determined

Computer programs: SNS EPICS, Mantid python program, *SHELXL2019*/3. No anomalous-dispersion-type corrections were applied; neutron scattering lengths were treated as real (software f”=0).

Table S4 Hydrogen-bond geometry (Å, °)

D—H···A	D—H	H···A	D···A	D—H···A
O13—H1···O10	0.965 (3)	1.773 (3)	2.6573 (14)	150.8 (3)
O12—H2···O4^{i}	0.974 (3)	1.746 (3)	2.7192 (14)	177.2 (3)
O11—H3···O2^{ii}	0.965 (3)	2.650 (3)	3.2430 (14)	120.1 (3)
O11—H3···O9^{iii}	0.965 (3)	1.833 (3)	2.7853 (14)	168.3 (3)
O13—H4···O7^{iv}	0.950 (3)	2.040 (4)	2.7576 (14)	130.9 (3)
O11—H5···O1	0.965 (3)	2.126 (3)	3.0520 (15)	160.2 (3)
O12—H6···O8^{v}	0.962 (3)	2.107 (3)	3.0499 (15)	166.2 (3)

Symmetry codes: (i) $x-1/2$, $y+1/2$, z; (ii) $x+1/2$, $-y+1/2$, $z+1/2$; (iii) x, y, $z+1$; (iv) x, $-y+1$, $z-1/2$; (v) $x-1/2$, $-y+1/2$, $z-1/2$.

S7.4. checkCIF validation loop and change log

S7.4.1. checkCIF status before validation

The initial CIF submission produced the following alert counts:

A = 6, B = 0, C = 5 G = 15 (recorded in the workflow checkCIF results view)

The dominant level A issues were missing required metadata tags, including:

EXPT005: missing _exptl_crystal_description

DIFF003: missing _diffrn_measurement_device_type

PLAT183: missing _cell_measurement_reflns_used

S7.4.2. checkCIF status after validation

After targeted metadata completion and CIF correction, the recorded checkCIF report excerpt indicates **0 level A and 0 level B alerts**, with remaining alerts at levels C and G. No level B alerts were present in either the initial or final report.

Table S5 checkCIF alert counts before and after validation.

Stage	Level A	Level B	Level C	Level G
Initial CIF (agent-guided checkCIF view)	6	0	5	15

Final validated CIF (checkCIF report excerpt)	0	0	4	14

"Initial" alert counts are taken from the agent-guided checkCIF panel. "Final" alert status and the remaining C-level items are taken from the checkCIF report excerpt for the validated CIF.

S7.4.3. checkCIF change log (alerts, actions, and outcomes)

The details, including content, modification, and outcome of checkCIF alerts addressed by NeuDiff agent are listed in Table S6.

Table S6 checkCIF change log for the validation loop (alerts, actions, and outcomes).

Alert ID	Level before	Issue summary	Action taken	CIF field(s) modified	Outcome
EXPT005	A	Missing crystal description	Added explicit crystal description from experiment context	_exptl_crystal_description	Resolved (no longer level A)
DIFF003	A	Missing measurement device type	Specified instrument / device type (TOPAZ)	_diffrn_measurement_device_type	Resolved (no longer level A)
PLAT183	A	Missing reflections used for cell measurement	Recorded reflections used for cell measurement	_cell_measurement_reflns_used	Resolved (no longer level A)
PLAT184	A	Missing minimum theta for cell measurement	Recorded minimum theta for cell measurement	_cell_measurement_theta_min	Resolved (no longer level A)
PLAT185	A	Missing maximum theta for cell measurement	Recorded maximum theta for cell measurement	_cell_measurement_theta_max	Resolved (no longer level A)

PLAT699	A	Missing crystal description	Added explicit crystal description from experiment context	_exptl_crystal_description	Resolved (no longer level A)
PLAT041	C	Calculated and reported Summed Formula differ	Change formula to IUCr alphabetical order convention	_chemical_formula_sum	Resolved (no longer level C)
PLAT883	G	The origin of primary solution for atom site is not provided	Add explicit information that the primary atom location is from X-ray measurement	_atom_sites_solution_primary	Resolved (no longer level G)

Items left at level C/G were retained only when they did not compromise the crystallographic interpretation and when the underlying generator produced values that were scientifically correct but conservatively formatted.

Reference:

Robertson, S. & Zaragoza, H. (2009). *INR* **3**, 333-389.

Wang, W., Wei, F., Dong, L., Bao, H., Yang, N. & Zhou, M. (2020). MiniLM: deep self-attention distillation for task-agnostic compression of pre-trained transformers. In Advances in Neural Information Processing Systems, Vol. 33, pp. 5776-5788. Curran Associates Inc.
